# Imperatorin Suppresses Anaphylactic Reaction and IgE-Mediated Allergic Responses by Inhibiting Multiple Steps of FceRI Signaling in Mast Cells: IMP Alleviates Allergic Responses in PCA

**DOI:** 10.1155/2019/7823761

**Published:** 2019-01-20

**Authors:** Zhemin Xian, Guangyu Jin, Hongmei Li, Jingzhi Jiang, Chongyang Wang, Lianhua Zhu, Zhehu Jin, Liangchang Li, Hongmei Piao, Mingyu Zheng, Guanghai Yan

**Affiliations:** ^1^Department of Respiratory Medicine, Affiliated Hospital of Yanbian University, Yanji 133000, Jilin, China; ^2^Department of Radiology, Yanbian University Hospital, YanJi 133002, Jilin, China; ^3^Administration of Traditional Chinese Medicine of Jilin Province, Changchun 130051, Jilin, China; ^4^Department of Anatomy, Histology and Embryology, Yanbian University Medical College, Yanji 133002, Jilin, China; ^5^Department of Dermatology, Yanbian University Hospital, Yanji City, Jilin, China; ^6^College of Pharmacy, Yanbian University, Yanji 133002, Jilin, China

## Abstract

This study is to investigate the effects of imperatorin (IMP) on allergic responses mediated by mast cells, both* in vitro* and* in vivo*. Passive cutaneous anaphylaxis (PCA) model was established. Histological detection was performed to assess the ear histology. ELISA and Western blot analysis were used to detect the levels of corresponding cytokines and signalling pathway proteins. IMP decreased the leakage of Evans blue and the ear thickness in the PCA models, in a dose-dependent manner, and alleviated the degranulation of mast cells. Moreover, IMP reduced the expression of TNF-*α*, IL-4, IL-1*β*, IL-8, and IL-13. Furthermore, IMP inhibited the phosphorylation levels of Syk, Lyn, PLC-*γ*1, and Gab2, as well as the downstream MAPK, PI3K/AKT, and NF-*κ*B signaling pathways. In addition, IMP inhibited the mast cell-mediated allergic responses through the Nrf2/HO-1 pathway. IMP attenuates the allergic responses through inhibiting the degranulation and decreasing the expression levels of proinflammatory cytokines in the mast cells, involving the PI3K/Akt, MAPK, NF-*κ*B, and Nrf2/HO-1 pathways.

## 1. Introduction

The morbidity of allergic diseases has been gradually increasing, and approximately 20% of the population suffers from allergic diseases, such as atopic anaphylaxis, dermatitis, allergic rhinitis, food allergy, and allergic asthma [1]. Allergic diseases are characterized by increased isotype switching of B cells and the T helper 2 cells, producing IgE antibodies against specific allergens [2]. It has been shown that the gathering of IgE and Fc*ε*RI on mast cells can trigger the allergic reaction. Therefore, the mast cells play pivotal roles in IgE-mediated allergic diseases [3]. After antigenic stimulation, the mast cells will secrete inflammatory mediators, such as histamine, proinflammatory cytokines, chemokines, eicosanoids, and proteases [4, 5]. These inflammatory mediators can activate the Src family kinases (Lyn and Syk), which motivates the calcium mobilization, as well as the phosphorylation of phosphatidylinositol 3-kinase (PI3K), Akt, mitogen-activated protein kinases (MAPKs), and nuclear factor (NF)-*κ*B [6, 7].

Among the inflammatory mediators, histamine is a prime factor in the acute IgE-mediated allergic diseases, which can increase the vasodilation and permeability of vessels, resulting in the hypothermia, edema, and leukocyte recruitment [8, 9]. At the late phase, the tumor necrosis factor (TNF)-*α*, interleukin (IL)-4, IL-8, IL-1*β*, and IL-13 would be liberated by the mast cells to cause the allergic inflammation [10, 11]. Therefore, decreasing the proinflammatory cytokine levels may relieve the allergic inflammatory symptoms.

Imperatorin (IMP) (9-(3-methylbut-2-enyloxy)-7H-furo[3,2-g]chromen-7-one) is the main component of the dried root or rhizome of* Radix Angelicae Dahuricae*. IMP has been shown to have versatile pharmacological effects, including the anti-inflammatory effect [12, 13]. A previous study has demonstrated that IMP decreases the inflammatory reaction by inhibiting the TNF-*α*-mediated activation of ROS/PI3K/Akt/NF-*κ*B pathway [14]. Moreover, IMP could also suppress the inflammation via the MAPK signaling pathway [15]. The signaling pathways of MAPK, PI3K/Akt, NF-*κ*B, and Nrf2 are the predominant cascades that regulate the expression of HO-1, which is closely related to the anti-inflammatory responses [16]. Previous studies have demonstrated that IMP could inhibit the degranulation and production of cyclooxygenase-2-dependent PGD2 and 5-lipoxygenase-dependent LTC4 via inhibiting the calcium mobilization and and blocking the phospholipase C*γ*1 and cytosolic phospholipase A2/MAPK/NF-*κ*B pathways, in the IgE-induced bone marrow-derived mast cells (BMMC) [13]. Therefore, IMP may have the effects in the treatment of allergic diseases through the anti-inflammatory and antioxidant functions. However, the detailed mechanisms for the IMP activities on the mast cell-mediated allergic responses are still unclear. In this study, the inhibitory effects of IMP on antiallergic effects related to the inhibition of mast cell degranulation and inflammatory cytokine expression were evaluated, both* in vitro* and* in vivo*.

## 2. Materials and Methods

### 2.1. Study Animals

The 7-week-old specific pathogen-free (SPF) inbred female BALB/c mice (n=50) and female Sprague-Dawley (SD) rats (n=20) were purchased from the House Section of Yanbian University Health Science Center (Yanji, Jilin, China). The animals were kept under standard laboratory conditions for 7 days prior to experiments and provided with water and standard chow* ad libitum*. All the animal experiments were approved by the Institutional Animal Care and Use Committee of Yanbian University School of Medical Sciences.

### 2.2. Preparation of Rat Peritoneal Mast Cells (RPMCs)

To obtain the RPMCs [17], the rats were anesthetized by ether and injected with 10 ml calcium-free N-(2-hydro-xyethyl) piperazine-N-2-ethanesulfonic acid (HEPES)-Tyrode buffer (137 mM NaCl, 5.6 mM glucose, 12 mM NaHCO_3_, 2.7 mM KCl, 0.3 mM NaH_2_PO_4_, and 0.1% gelatin) into the peritoneal cavity, and the abdominal region was mildly kneaded for 90 s. The peritoneal lavage fluid was collected with a Pasteur pipette, and RPMCs were cleaned by the Percoll (Pharmacia, Uppsala, Sweden) [18]. The purity of the prepared RPMCs was detected by the toluidine blue staining. Purified mast cells (1×10^6^ cells/ml) were resuspended in the HEPES-Tyrode buffer.

### 2.3. MTT Assay

RBL-2H3 cells and RPMCs (2×10^4^/well in the 96-well plates) were treated with indicated concentrations of IMP for 24 h and then incubated with 1 mg/ml MTT at 37°C for 2 h. DMSO was added and the absorbance at 570 nm was detected using a spectrophotometer (Spectra MAX PLUS; Molecular Devices, Sunnyvale, CA, USA).

### 2.4. Cell Culture

IMP (MAYA-CR-6162; purity>98%) was purchased from the Maya Reagent (Jiaxing, Zhejiang, China). RBL-2H3 cells and RPMCs were cultured with DMEM containing 10% FBS, supplemented with 100 U/ml penicillin G and 100 *μ*g/ml streptomycin, in a 37°C, 5% CO_2_ incubator. The cells were divided into the following five groups: the control (Con), IgE+Ag, IgE+Ag+IMP (10*μ*M), IgE+Ag+IMP (20*μ*M), and IgE+Ag+IMP (40*μ*M) groups. Cells in the Con group were cultured without any drug intervention. Cells in the IgE+Ag group were challenged with 10 *μ*g/ml anti-DNP IgE (Sigma-Aldrich Chemical Co., St. Louis, MO, USA) for 6 h and treated with DNP-HSA (Sigma-Aldrich Chemical Co.) (100 ng/ml) for 10 min. In the IgE+Ag+IMP groups, IMP (10, 20, and 40 *μ*M, respectively) was used to incubate the cells at 37°C for 30 min, before challenged with DNP-HSA (100 ng/ml).

### 2.5. Passive Cutaneous Anaphylaxis (PCA) Test and Ear Swelling Response

Mice were divided into the control (Con), IgE+Ag, IgE+Ag+IMP (15 mg/kg), IgE+Ag+IMP (30 mg/kg), and IgE+Ag+IMP (60 mg/kg) groups (n=10 per group), respectively. Mice in the Con group accepted the same volume of phosphate-buffered saline (PBS). Mice in the IgE+Ag group were intradermally sensitized by 200 ng anti-DNP IgE in 20 *μ*l PBS in the dorsal skin on the right, and a sham PBS injection was given in the left dorsal skin. After 24 h, each mouse was injected via the tail vein with 1% Evans blue (Sigma-Aldrich Chemical Co.) and 0.1 mg antigen (DNP-HSA). In the IgE+Ag+IMP group, at 1 h before the antigen challenge, IMP (15, 30, and 60 mg/kg of body weight, respectively) was administered intraperitoneally. After 30 min, the mice were sacrificed. The weight of the pigment area at the injection site was measured. The removed skin was incubated with formamide at 55°C for 24 h, and then the extravasated Evans blue dye was extracted. The amount of dye absorbance at 620 nm was measured with the Spectra MAX PLUS spectrophotometer (Molecular Devices). The section was stained with toluidine blue, and the number of mast cells was counted under 100× magnification. For the assessment of ear swelling response, the mice were anesthetized by intraperitoneal injection of 50 *μ*l mixture (1:1) of ketamine (1 mg/ml) and xylazine hydrochloride (23.32 mg/ml), and the ear thickness was measured with a digital micrometer (Kawasaki, Japan)

### 2.6. Histological Analysis, Mast Cell, and Mast Cell Degranulation Counting

After fixation and embedment, the ears were cut into 5 *μ*m sections. The toluidine blue and H&E staining was conducted, and the number of mast cells was counted in randomly selected five fields, under 100× magnification. The number of mast cell degranulation was counted in 5 randomly selected fields (1000× magnification).

### 2.7. Histamine Content Measurement

The histamine contents were measured by the radioenzymatic method. RBL-2H3 cells and RPMCs were challenged with DNP-HAS (100 ng/ml) before preincubating with IMP at 37°C for 30 min. After centrifugation at 150 ×g at 4°C for 10 min, the supernatant was harvested to measure the histamine contents.

### 2.8. Measurement of  ^45^*Ca* Uptake

The measurement of ^45^Ca uptake was performed according to a previously published method [ [17]]. RPMCs were resuspended in HEPES-Tyrode buffer containing 45Ca (1.5 mCi/ml; Perkin-Elmer Life Sciences, MA, USA) at 4 °C for 10 min. Mast cell suspensions were challenged with 10 *μ*g/ml anti-DNP IgE for 6 h and preincubated with various concentrations of IMP. The reaction was proceeded at 37°C for 30 min prior to challenge with DNP-HAS (100 ng/ml), which was terminated by the addition of 1 mM lanthanum chloride. The samples were centrifuged at 150 ×g for 10 min for 3 times, and then the mast cells were lysed with 10% Triton X-100. The radioactivity was measured with a Liquid Scintillation Analyzer (A Canberra Company, Australia).

### 2.9. Enzyme-Linked Immunosorbent Assay (ELISA)

The contents of TNF-*α*, IL-4, IL-1*β*, IL-8, and IL-13 were measured with the commercially available kits. For the measurement, the supernatant of treated RBL-2H3 cells was collected, which were then subjected to the assessment with the TNF-*α*, IL-4, IL-1*β*, and IL-13 ELISA kits (Abcam, Cambridge, MA, UK) and the IL-8 ELISA kit (Cusabio, Wuhan, Hubei, China), respectively. The absorbance at 450 nm was measured.

### 2.10. Western Blot Analysis

Total protein was extracted from the lung tissue. The protein concentration was determined by the BCA kit (Beyotime, Hunan, Hubei, China). Equal quantity of protein samples was separated by SDS-PAGE and then transferred onto the nitrocellulose membrane. The membrane were blocked with 5% skimmed milk and then incubated with primary antibodies at 4°C overnight. Then the membrane was incubated with horseradish peroxidase-conjugated goat anti-rabbit secondary antibody (#5151; 1:2000 dilution; Cell Signaling, Beverly, MA, USA) at room temperature for 2 h. Immunodetection was developed with the ECL detection reagent (Beyotime). Photographs were taken and the optical densities of protein bands were scanned and quantified with the Gel Doc 2000 software (Bio-Rad, Hercules, CA, USA). The antibodies of I*κ*B*α* (#4814; 1:1000 dilution), phospho-I*κ*B*α* (#2859; 1:1000 dilution), NF-*κ*B p65 (#8242; 1:1000 dilution), Akt (#2920; 1:1000 dilution), phospho-Akt (#9611; 1:1000 dilution), ERK (#4695; 1:1000 dilution), phospho-ERK (#4370; 1:1000 dilution), p38 (#8690; 1:1000 dilution), phospho-p38 (#4511; 1:1000 dilution), JNK (#9253; 1:1000 dilution), phospho-JNK (#9255; 1:1000 dilution), Nrf2 (#12721; 1:1000 dilution), HO-1 (#82206; 1:1000 dilution), Syk (#13198; 1:1000 dilution), phospho-Syk (#2710; 1:1000 dilution), Lyn (#2796; 1:1000 dilution), phospho-Lyn (#2731; 1:1000 dilution), Gab2 (#3239; 1:500 dilution), phospho-Gab2 (#3881; 1:500 dilution), PLC*γ*1 (#2822; 1:1000 dilution), phospho-PLC*γ*1 (#8713; 1:1000 dilution), and anti-GAPDH (#2118; 1:1000 dilution) were all purchased from Cell Signaling Technology (Beverly, MA, USA).

### 2.11. Statistical Analysis

Data were expressed as mean ± SD. Statistical analysis was performed using the Prism 6.00 software (GraphPad Software, San Diego, CA, USA). All the data followed the normal distribution as analyzed by the Kolmogorov-Smirnov test. One-way analysis of variance was conducted for the multiple comparisons, followed by the Dunnett post hoc test.* P*<0.05 was considered statistically significant.

## 3. Results

### 3.1. IMP Decreases Anti-DNP IgE-Mediated PCA

The effects of IMP on the PCA mouse model were analyzed by the assessment of the local extravasation of Evans blue dye. Our results showed that, compared with the Con group, the levels of dye extravasation were greatly higher in the IgE+Ag group. Moreover, the IMP (15, 30, and 60 mg/kg of body weight) decreased the dye extravasation, in a dose-dependent manner (Table 1). Compared with the IgE+Ag group, IMP (30 and 60 mg/kg) significantly reduced the dye extravasation. These results suggest that IMP could decrease the anti-DNP IgE-mediated PCA in mouse models.

### 3.2. IMP Alleviates Anti-DNP IgE-Mediated Ear Swelling

**Figure 1:**
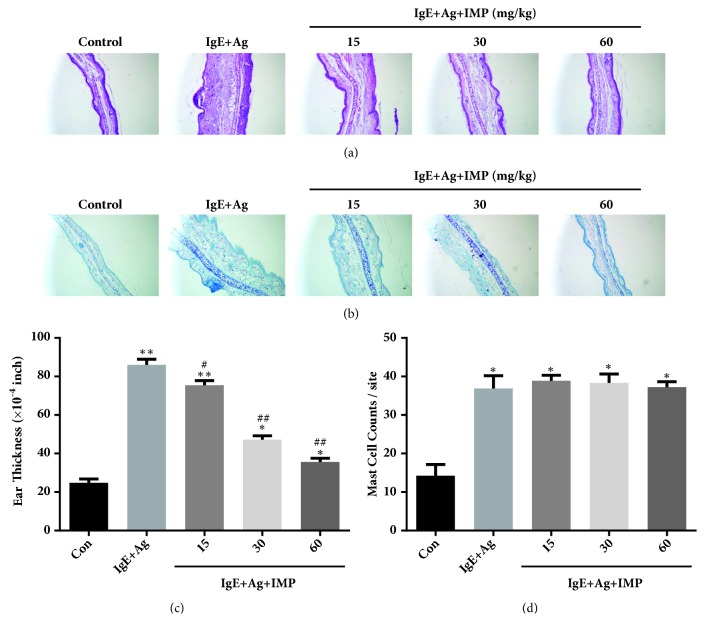
IMP alleviated the anti-DNP IgE-mediated ear histology and ear swelling. Mice were divided into the Con, IgE+Ag, and IgE+Ag+IMP (15, 30, and 60mg/kg) groups. (a-b) Ear section was stained with the H&E and toluidine blue staining (200× magnification). (c) Ear thickness was measured with the dial thickness gauge. (d) The number of mast cells at the dermis was counted. Compared with the Con group, ^∗^*P*<0.05, ^∗∗^*P*<0.01; compared with the IgE+Ag group, ^#^*P*<0.05, ^##^*P*<0.01.

The effects of IMP on the anti-DNP IgE-induced ear histology were detected by the H&E and toluidine blue staining. As shown in Figure 1(a), the IgE+Ag group represented remarkably thicker ears than the Con group, and the ear skin dermis had inflammatory cells infiltration and hypodermis edema. Nevertheless, IMP inhibited the pathological changes, in a dose-dependent manner. Moreover, IMP also decreased the ear swelling and thickness (Figure 1(c)). Compared with the Con group, the IgE+Ag group was associated with significantly increased mast cells in the ear, but the IMP administration had no apparent effects on the mast cell number (Figures 1(b) and 1(d)). The results imply that IMP decreases the inflammatory responses and inflammatory swelling by inhibiting the activation of mast cells in the anti-DNP IgE-mediated allergic responses.

### 3.3. IMP Reduces Degranulation, Histamine Release, and Intracellular Calcium Level of Mast Cells

**Figure 2:**
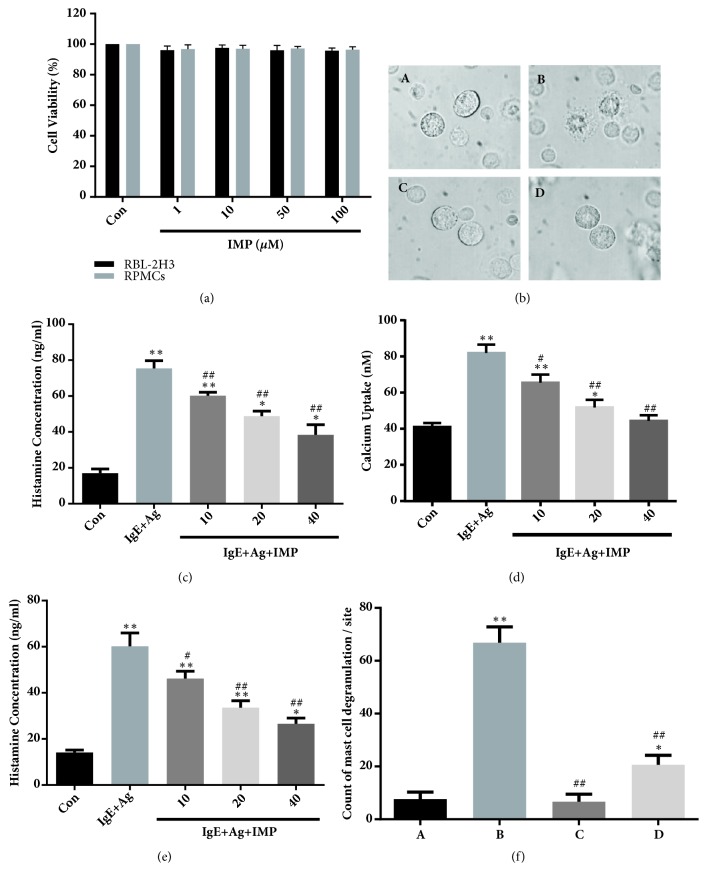
IMP reduced degranulation, histamine release, and intracellular calcium level of mast cells. (a) RBL-2H3 cells and purified RPMCs were treated with indicated concentrations of IMP. Cell viability was measured by MTT assay. (b) Effects of IMP on the degranulation of RPMCs (1000× magnification). (A) Normal RPMCs in HEPES-Tyrode buffer; (B) RPMCs sensitized with 10 *μ*g/ml anti-DNP IgE for 6 h and challenged with 100 ng/ml DNP-HAS; (C) RPMCs incubated with 40 *μ*M IMP at 37°C for 30 min; (D) Prior to the challenge with 100 ng/ml DNP-HAS, preincubated with 40 *μ*M IMP at 37°C for 30min. (c) RBL-2H3 cells were stimulated with 10 *μ*g/ml anti-DNP IgE for 6 h and challenged with 100 ng/ml DNP-HAS. The histamine release of RBL-2H3 cells was measured by the radioenzymatic method. (d) RPMCs were stimulated with 10 *μ*g/ml anti-DNP IgE for 6 h and challenged with 100 ng/ml DNP-HAS. Calcium uptake was measured by the radioenzymatic method. (e) RPMCs were stimulated with 10 g/ml anti-DNP IgE for 6 h and challenged with 100 ng/ml DNP-HAS. The histamine release of RPMCs was measured by the radioenzymatic method. (f) Results of quantitative analysis of mast cell degranulation. Compared with the Con group, ^∗^*P*<0.05, ^∗∗^*P*<0.01; compared with the IgE+Ag group, ^#^*P*<0.05, ^##^*P*<0.01.

The MTT assay was used to measure the effects of IMP on the cell viability. RBL-2H3 cells and RPMCs were pretreated with indicated concentrations of IMP (i.e., 1-100 *μ*M) for 24 h. Our results showed that IMP (up to 100*μ*M) almost had no influence on the viability of RBL-2H3 cells and RPMCs (Figure 2(a)). However, IMP obviously decreased the degranulation of RPMCs (Figures 2(b) and 2(f)). Moreover, IMP also reduced the histamine release of RBL-2H3 cells and RPMCs, in a dose-dependent manner (*P*< 0.01) (Figures 2(c) and 2(e)).

To prove the mechanism of IMP-induced reduction of histamine release, the intracellular calcium levels were detected. The levels of intracellular calcium in the RPMCs were increased when exposed to DNP-HSA; however, IMP decreased the intracellular calcium levels, in a dose-dependent manner (*P*< 0.05) (Figure 2(d)). The results suggest that IMP could reduce degranulation and histamine release, as well as the intracellular calcium level, in the mast cells.

### 3.4. Effects of IMP on Inflammatory Cytokine Release

**Figure 3:**
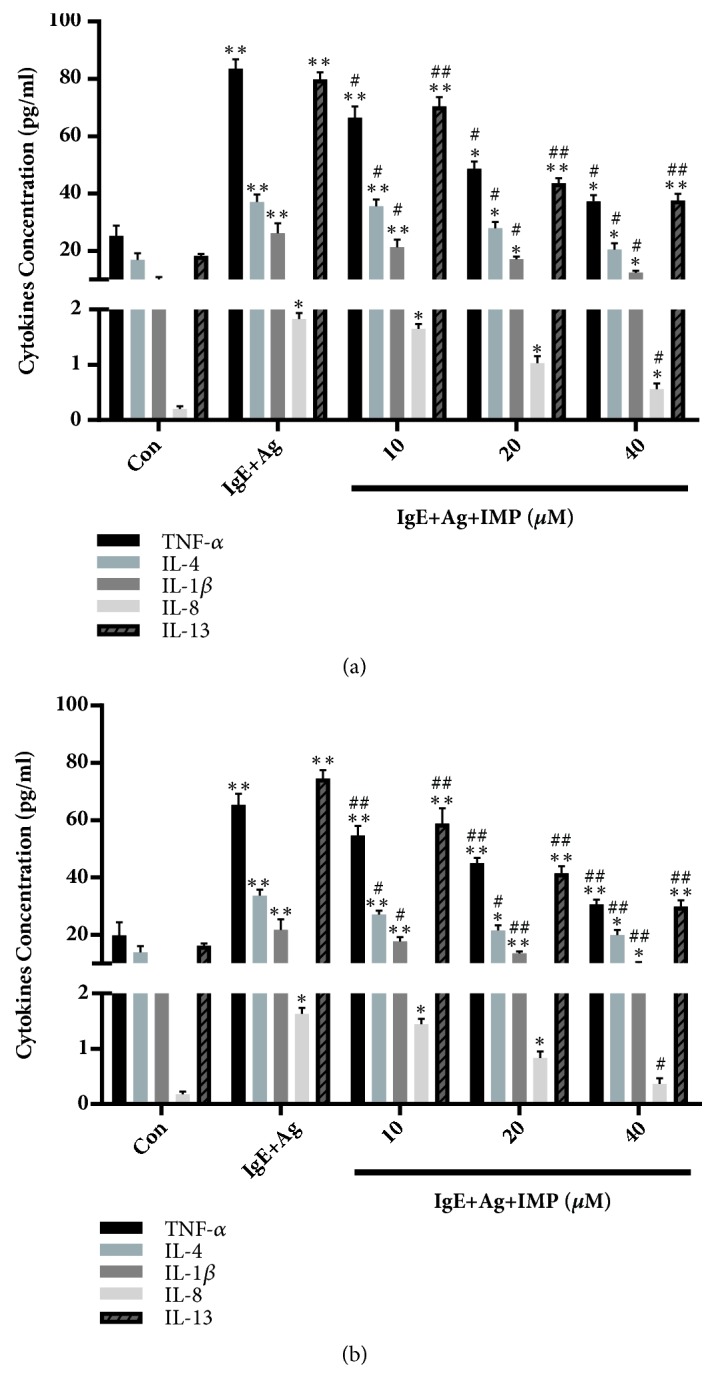
Effects of IMP on inflammatory cytokine release in RBL-2H3 cells and RPMCs. (a) RBL-2H3 cells and (b) RPMCs were divided into the Con, IgE+Ag, and IgE+Ag+IMP groups. They were stimulated with 10 *μ*g/ml anti-DNP IgE for 6 h and challenged with 100 ng/ml DNP-HAS, in the absence or presence of IMP (10, 20, and 40 *μ*M, respectively). The levels of TNF-*α*, IL-1*β*, IL-4, IL-8, and IL-13 in the culture supernatant were detected by ELISA. Compared with the Con group, ^∗^*P*<0.05, ^∗∗^*P*<0.01; compared with the IgE+Ag group, ^#^*P*<0.05, ^##^*P*<0.01.

Activation of mast cells could promote the release of inflammatory cytokines. Therefore, the effects of IMP on the release of TNF-*α*, IL-8, IL-1*β*, IL-4, and IL-13 in the RBL-2H3 cells and RPMCs were detected with ELISA. Our results showed that the levels of cytokines were greatly increased in the IgE+Ag group, compared with the Con group (Figures 3(a) and 3(b)). Nevertheless, IMP significantly reduced the cytokine secretions at the concentrations of 20 and 40 *μ*M, in the RBL-2H3 cells and RPMCs (Figures 3(a) and 3(b)). These results suggest that IMP may inhibit the secretion of inflammatory cytokine in the mast cells.

### 3.5. Effects of IMP on Fc*ε*RI-Mediated Signaling Pathway in RBL-2H3 Cells

**Figure 4:**
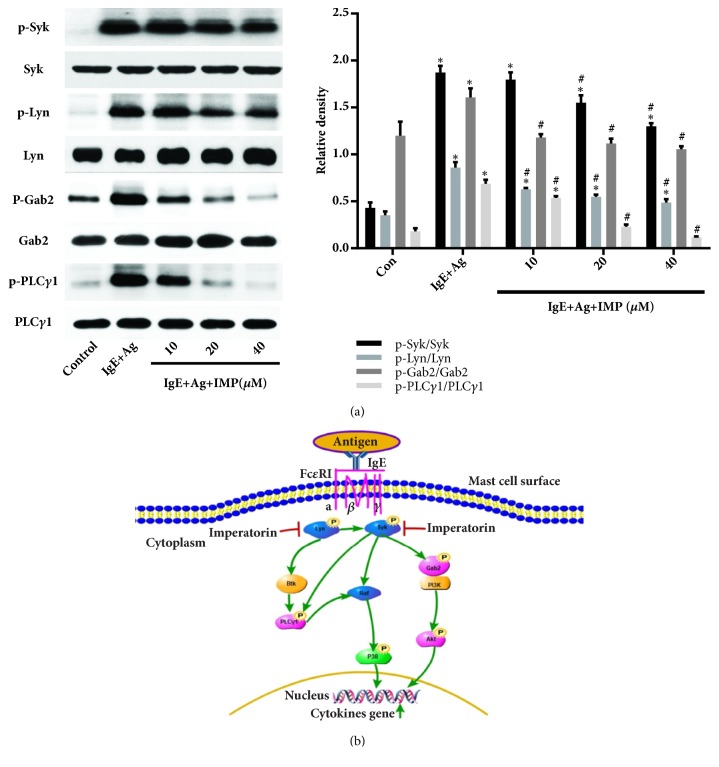
Effects of IMP on Fc*ε*RI-mediated signaling pathway in RBL-2H3 cells. RBL-2H3 cells were divided into the Con, IgE+Ag, and IgE+Ag+IMP groups. RBL-2H3 cells were stimulated with 10 *μ*g/ml anti-DNP IgE for 6 h and challenged with 100 ng/ml DNP-HAS, in the absence or presence of IMP (10, 20, and 40 *μ*M, respectively). Levels of Syk, Lyn, Gab2, and PLC-*γ*1were detected by Western blot analysis. Compared with the Con group, ^∗^*P*<0.05, ^∗∗^*P*<0.01; compared with the IgE+Ag group, ^#^*P*<0.05, ^##^*P*<0.01. (b) Illustration on the possible relationship between IMP and Fc*ε*RI-mediated signaling pathway. IMP disabled the phosphorylation of Syk, Lyn, Gab2, and PLCg1. We demonstrated that IMP suppressed the allergic inflammation mediated by mast cells, and we suppose that this effect may be mediated by the Fc epsilon RI-dependent signaling pathway.

To investigate the influences of IMP on signaling pathways mediated by Fc*ε*RI, the key proteins of Syk, Lyn, Gab2, and PLC-*γ*1 were detected by Western blot analysis. Our results showed that, compared with the Con group, the expression levels of p-Syk, p-Lyn, p-Gab2, and p-PLC-*γ*1 were greatly increased in the IgE+Ag group (*P*<0.05). Compared with the IgE+Ag group, IMP remarkably decreased the phosphorylation levels of Syk, Lyn, and Gab2, and the expression level of downstream molecular PLC-*γ*1, in a dose-dependent manner (*P*<0.05) (Figure 4). These results suggest that IMP downregulates the Fc*ε*RI-mediated signaling pathway in RBL-2H3 cells.

### 3.6. Effects of IMP on Phosphorylation of Akt, ERK, p38, and JNK in RBL-2H3 Cells

**Figure 5:**
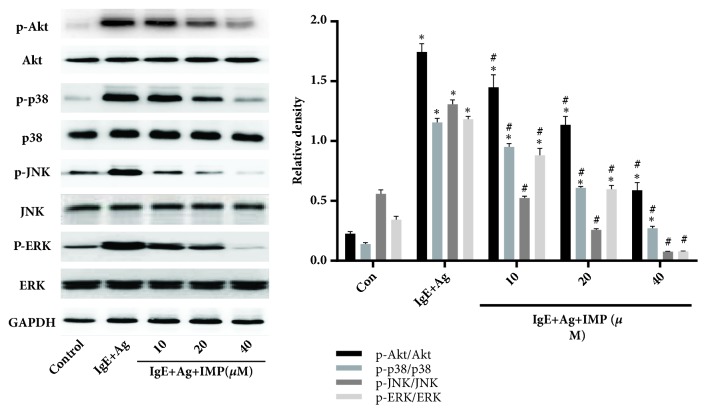
Effects of IMP on activation of AKT and MAPK signaling pathways in RBL-2H3 cells. RBL-2H3 cells were divided into the Con, IgE+Ag, and IgE+Ag+IMP groups. RBL-2H3 cells were stimulated with 10 *μ*g/ml anti-DNP IgE for 6 h and challenged with 100 ng/ml DNP-HAS, in the absence or presence of IMP (10, 20, and 40 *μ*M, respectively). Levels of Akt, p38, JNK, and ERK were detected by Western blot analysis. Compared with the Con group, ^∗^*P*<0.05, ^∗∗^*P*<0.01; compared with the IgE+Ag group, ^#^*P*<0.05, ^##^*P*<0.01.

The expression levels of Akt, p38, JNK, and ERK were then detected by Western blot analysis. Our results showed that, compared with the Con group, the phosphorylation levels of Akt, p38, ERK, and JNK were greatly increased in the IgE+Ag group (*P*<0.05), but these pathways were inhibited by IMP, in a dose-dependent manner (Figure 5). These results suggest that IMP may play an important role in the antiallergic function through inhibiting the MAPK and Pi3k/Akt signaling pathways. These results implied that IMP downregulated the activation of Pi3k/Akt and MAPK signaling pathway.

### 3.7. Effects of IMP on Activation of NF-*κ*B and Nrf2/HO-1 Signaling Pathways

**Figure 6:**
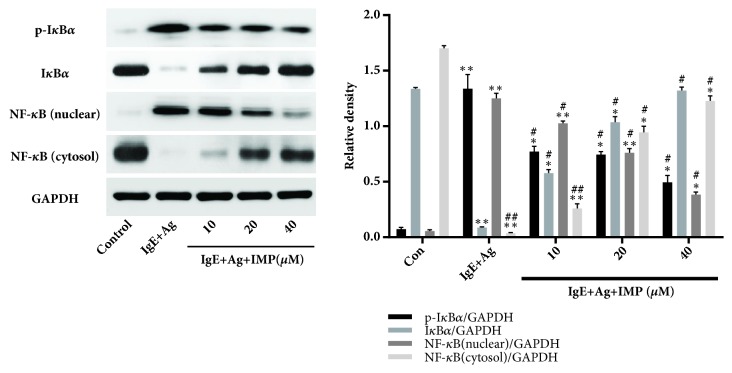
Effects of IMP on activation of NF-*κ*B signaling pathway in RBL-2H3 cells. RBL-2H3 cells were divided into the Con, IgE+Ag, and IgE+Ag+IMP groups. RBL-2H3 cells were stimulated with 10 *μ*g/ml anti-DNP IgE for 6 h and challenged with 100 ng/ml DNP-HAS, in the absence or presence of IMP (10, 20, and 40 *μ*M, respectively). The key proteins of NF-*κ*B signaling pathway were detected by Western blot analysis. Compared with the Con group, ^∗^*P*<0.05, ^∗∗^*P*<0.01; compared with the IgE+Ag group, ^#^*P*<0.05, ^##^*P*<0.01.

**Figure 7:**
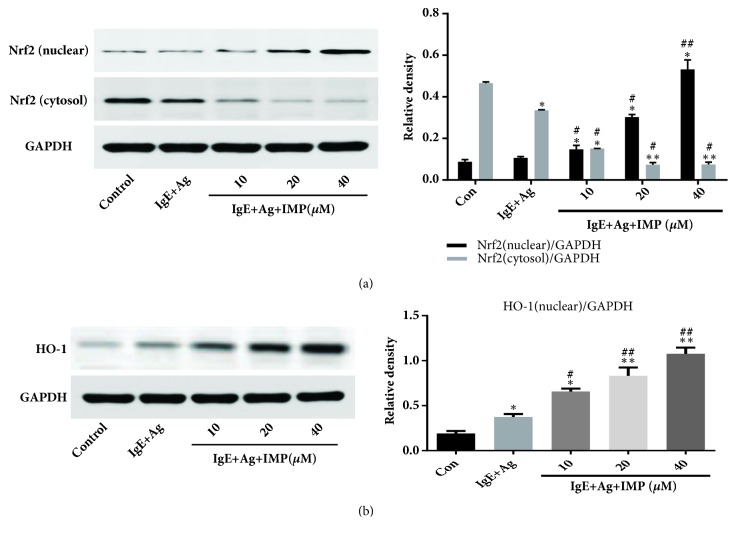
Effects of IMP on activation of Nrf2 and HO-1 signaling pathways. RBL-2H3 cells were stimulated with 10 *μ*g/ml anti-DNP IgE for 6 h and challenged with 100 ng/ml DNP-HAS, in the absence or presence of IMP (10, 20, and 40 *μ*M, respectively). (a-b) Nrf2 (a) and HO-1 (b) levels were detected by Western blot analysis. Compared with the Con group, ^∗^*P*<0.05, ^∗∗^*P*<0.01; compared with the IgE+Ag group, ^#^*P*<0.05, ^##^*P*<0.01.

The effects of IMP on activation of NF-*κ*B and Nrf2/HO-1 signaling pathways were then investigated. Our results showed that IMP decreased the expression of NF-*κ*B p65 in the nuclear and increased its expression in the cytosol. Moreover, IMP significantly decreased the phosphorylation level of I*κ*B*α* (Figure 6). These results suggest that IMP greatly inhibits the NF-*κ*B p65 shift into the nuclear. Moreover, the Nrf2 expression levels (nuclear) in the IgE+Ag and IMP groups were significantly higher than the Con group (both* P*<0.01), but the IMP groups had greatly higher levels of Nrf2 (nuclear) than the IgE+Ag group (*P*<0.01). Contrary results were obtained for the expression of Nrf2 in the cytosol. Furthermore, the expression levels of HO-1 in the IgE+Ag and IMP groups were significantly higher than the Con group (all* P*<0.05), and the IMP groups had significantly higher levels of HO-1 than the IgE+Ag group, in a dose-dependent manner (*P*<0.05) (Figure 7). These results suggest that IMP downregulates the activation of NF-*κ*B and upregulates the activation of Nrf2/HO-1 signaling pathways.

## 4. Discussion

In recent years, a lot of natural products have been discovered as new drugs. IMP is a Chinese medicinal ingredient of coumarins. It has been shown that IMP has several pharmacological effects, such as anti-inflammatory, anticonvulsant, antitumoral, antihypertensive, vasodilatation, and antihypertrophic effects [12, 19–21]. Administration of the single, effective compound could enhance the bioavailability and decrease the side effects. In this study, the antiallergic inflammatory effects of IMP were investigated, both* in vitro* and* in vivo*.

In this study, the local allergic responses mediated by mast cells are assessed with the PCA model [22]. The results showed that IMP markedly decreased the ear thickness and the leakage of Evans blue in the PCA models. Nevertheless, IMP alleviated the local allergic responses with the exception of the numbers of mast cells. These results suggest that IMP inhibits the allergic responses mediated by mast cells, but IMP could not influence the quantity of mast cells.

Degranulation of mast cells is activated by the IgE and the numerous secreted mediators, which play pivotal roles in the allergic responses [22]. Thus, the suppression of mast cell degranulation might be crucial to alleviate the allergic responses. Previous studies have found that intracellular calcium levels of mast cells would regulate the degranulation [22–24]. After they were activated by agents, the intracellular calcium levels of mast cells would be obviously increased [25, 26]. The results suggest that IMP can not only significantly suppress the degranulation of RPMCs, but also decrease the histamine release and intracellular calcium level, in a dose-dependent manner. Therefore, IMP may inhibit the degranulation of IgE-mediated mast cells and the histamine release via reducing the intracellular calcium levels.

TNF-*α*, IL-4, IL-8, and IL-1*β* are representative proinflammatory cytokines playing key roles in the allergic inflammation [27]. TNF-*α* can promote the inflammation effusion [28, 29]. IL-4 and IL-13 are essential for the allergic reactions, which can promote the production of IgE in the plasma B cells [30]. IL-8 and IL-1*β* can induce the inflammatory cell activation and transmigration [31]. Thus, the inflammatory and allergic responses are closely related. Inhibiting the production of proinflammatory cytokine may suppress the allergic responses. Our results from the ELISA suggested that IMP decreased the levels of TNF-*α*, IL-1*β*, IL-4, IL-8, and IL-13 in the antigen-induced RBL-2H3 cells. Previous studies have showed that NF-*κ*B can promote the production of cytokines in the allergic inflammation [32, 33]. These results suggest that IMP suppress the transfer of NF-*κ*B into the nucleus, under challenging conditions. Therefore, IMP may inhibit the production of proinflammatory cytokines via regulating the NF-*κ*B signaling pathway.

Syk can induce the activation of mast cells, and the interaction of Fc*ε*RI and other factors (Lyn and Fyn) could regulate the Syk and downstream signals [34, 35]. Syk regulates the production of inflammatory cytokines through activating the downstream molecules, such as PLC-*γ*1 and Gab2 [36]. To confirm the effects of IMP on mast cells, the protein of Syk, Lyn, PLC-*γ*1, and Gab2 was measured. Our results showed that IMP decreased the phosphorylation levels of Syk, Lyn, PLC-*γ*1, and Gab2 induced by antigens. Previous studies have shown that PLC/PKC are related to the activation of MAPK and NF-*κ*B, and the phosphorylation of Gab2 can promote the expression of the PI3K/Akt pathway [37, 38]. The activation of MAPK and PI3K/Akt signaling pathways can induce the expression of inflammatory cytokines and degranulation in the mast cells [39]. The results showed that IMP decreased the expression levels of Akt and MAPK, in a dose-dependent manner. These results suggest that IMP inhibits the Fc*ε*RI-mediated signaling pathways to alleviate the allergic responses.

Previous studies have demonstrated that the inflammatory reaction would be influenced by the Nrf2/HO-1 pathways [40, 41]. Nrf2 can negatively regulate the inflammatory responses and there is a cross-talk between the Nrf2 and NF-*κ*B in the inflammatory responses [42, 43]. Furthermore, Nrf2 is phosphorylated by the MAPK and PI3K/Akt pathways, which promotes the release from the Keap1-mediated repression [44]. The results showed that IMP increased the expression of Nrf2 and HO-1, in a dose-dependent manner. These results suggest that IMP has antioxidation effects, which may inhibit the mast cell-mediated allergic responses, through the Nrf2/HO-1 pathways.

## 5. Conclusions

Our results showed that IMP could decrease the ear thickness and the leakage of Evans blue in the PCA models and inhibit the degranulation and inflammatory cytokine secretion in the RBL-2H3 mast cells stimulated by antigens. IMP also inhibited the phosphorylation of Syk, Lyn, PLC-*γ*1, and Gab2, as well as the downstream MAPK, PI3K/AKT, and NF-*κ*B signaling pathways. Moreover, IMP inhibited the mast cell-mediated allergic responses through the Nrf2/HO-1 pathways. Our findings suggest that IMP is a potential therapeutic drug for the allergic diseases via inhibiting the degranulation and decreasing the proinflammatory cytokine expression in the mast cells.

## Figures and Tables

**Table 1:** IMP alleviated anti-DNP IgE-mediated PCA.

Group	Amount of Evans blue (*μ*g/g)

Con	47.58±3.22
IgE+ Ag	268.67±8.62^∗^
IgE+Ag+IMP (15mg/kg BW)	235.33±6.69^∗^
IgE+Ag+IMP (30mg/kg BW)	182.58±8.65^#^
IgE+Ag+IMP (60mg/kg BW)	150.26±10.11^##^

Note: compared with the Con group, ^∗^*P*<0.05; compared with the IgE+Ag group, ^#^*P*<0.05, ^##^*P*<0.01. BW, body weight.

## Data Availability

The data used to support the findings of this study are available from the corresponding author upon request.
